# Sleeping Beauty transposase structure allows rational design of hyperactive variants for genetic engineering

**DOI:** 10.1038/ncomms11126

**Published:** 2016-03-30

**Authors:** Franka Voigt, Lisa Wiedemann, Cecilia Zuliani, Irma Querques, Attila Sebe, Lajos Mátés, Zsuzsanna Izsvák, Zoltán Ivics, Orsolya Barabas

**Affiliations:** 1 European Molecular Biology Laboratory, Structural and Computational Biology Unit, Meyerhofstrasse 1, Heidelberg 69117, Germany; 2 Paul Ehrlich Institute, Division of Medical Biotechnology, Paul Ehrlich Strasse 51-59, Langen 63225, Germany; 3 Max Delbrück Center for Molecular Medicine, Robert Rössle Strasse 10, Berlin 13092, Germany

## Abstract

*Sleeping Beauty* (*SB*) is a prominent *Tc1/mariner* superfamily DNA transposon that provides a popular genome engineering tool in a broad range of organisms. It is mobilized by a transposase enzyme that catalyses DNA cleavage and integration at short specific sequences at the transposon ends. To facilitate *SB*'s applications, here we determine the crystal structure of the transposase catalytic domain and use it to model the SB transposase/transposon end/target DNA complex. Together with biochemical and cell-based transposition assays, our structure reveals mechanistic insights into *SB* transposition and rationalizes previous hyperactive transposase mutations. Moreover, our data enables us to design two additional hyperactive transposase variants. Our work provides a useful resource and proof-of-concept for structure-based engineering of tailored SB transposases.

## Abstract (web summary)

Sleeping Beauty is used as a genome engineering tool in a range of organisms. Here, the authors solve an atomic structure of Sleeping Beauty (SB) transposase and model the target DNA into the active site, elucidating details that may enable the rational design of novel transposases.

## Graphical abstract

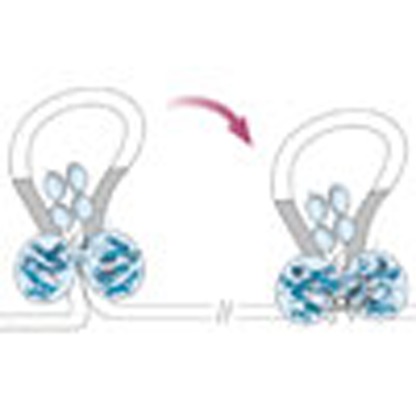

Transposons are natural DNA-mobilizing vehicles that have an intrinsic ability for genomic integration. In contrast to other genome engineering tools, such as zinc-finger nucleases, TALENs or the CRISPR/Cas9 system, transposons directly insert their genetic cargo into genomes, and can thereby enable stable gene transfer with high efficiency potentially alleviating the need for clonal selection in medical applications. *SB* is a member of the widespread *Tc1/mariner* superfamily of DNA transposons that has been developed as a genome engineering tool in a broad range of organisms^1^,^2^. It offers up to 95% gene transfer efficiency in diverse vertebrate cell types and is widely used in forward genetic screens^3^,^4^ as well as in *ex vivo* human gene therapy trials^5^,^6^. The transposon (tnp) DNA includes terminal inverted repeats (TIRs) at its ends and encodes a transposase protein, the workhorse of transposition, that catalyses all DNA cleavage and joining reactions required for transposition. Structural and biochemical studies of model *Tc1/mariner* transposons (*Tc3*, *Mos1* and *HsMar1*)^7^,^8^,^9^ revealed a so-called cut-and-paste mechanism that involves: (i) transposase binding to the TIRs; (ii) synapsis of the tnp ends generating an intertwined nucleoprotein complex, the paired-end complex (PEC) (also called transpososome); (iii) coordinated stepwise cleavage of the two DNA strands on both tnp ends; (iv) target DNA (tDNA) binding via recognition of a TA dinucleotide; and (v) integration into the new genomic location. Integration occurs with a two-base-pair (bp) stagger at each side of the target TA and results in single-strand gaps on both transposon flanks that are repaired subsequently by host enzymes leading to characteristic target site duplication.

Tc1/mariner transposases contain an N-terminal bipartite PAIRED-like^10^ DNA-binding domain (DBD) consisting of two helix-turn-helix motifs, and a C-terminal catalytic domain with an RNaseH-like fold and a catalytic triad of three acidic residues (DDE)^1^ that execute DNA hydrolysis (in excision) and transesterification (in integration) in a two metal ion-dependent manner^11^,^12^. Well-studied Tc1/mariner transposases (for example, Mos1 and HsMar1) function as homodimers that likely undergo multiple conformational rearrangements throughout the transposition reaction^7^,^8^,^13^,^14^,^15^. They initially assemble as auto-inhibited dimers, which rearrange upon DNA binding to allow hydrolysis of the first DNA strand (non-transferred strand, NTS) 2–3 nucleotides (nt) inside the tnp on both ends^8^,^14^. Subsequently, the transpososome changes conformation to bring the second DNA strand (transferred strand, TS) into the transposase active site^7^,^8^,^15^ for cleavage at the exact tnp boundary. Finally, tDNA capture might induce further conformational changes, but since the post-TS-cleavage transpososome already holds the terminal 3′OH groups of the TS in the transposase active sites, no major rearrangements seem to be required to proceed to integration. From all the distinct nucleoprotein assemblies, high-resolution structural information is so far only available for the *Drosophila mauritiana* Mos1 pre-16 and post-TS-cleavage^7^ PECs, both of which contain the TS ends in the transposase active sites. These highly similar structures depict the tnp ends in a parallel arrangement with the 3′OH ends of the TS-s situated 24.8 Å from each other. This arrangement appears consistent with a concerted attack of the two tnp ends on the tDNA with a 2-bp stagger^7^, although the 3′OH groups are somewhat far, suggesting that the tDNA may need to be bent for efficient integration. In accordance, bent tDNA has been shown to be a preferred target for integration of *Tc1/mariners*^17^,^18^,^19^, including *SB*^20^,^21^ and might even be a general feature of DDE enzymes^22^,^23^.

The 39 kDa SB transposase (340 amino acids (aa)) features a similar domain composition to other Tc1/mariner transposases. However, it has limited (<20%) sequence identity to its relatives and includes sub-domains with unknown functions, such as the glycine-rich strip^1^ (aa 183–198) that is specific to the Tc1 subfamily at the base of the so-called clamp loop (aa 159–190)^7^. *SB*'s transposon end architecture is also unusual: while the *mariner* transposons *HsMar1* and *Mos1* contain short 30–40 bp almost perfect inverted repeats, *SB*'s TIRs are ∼230 bp long and contain two 30–35 bp highly conserved transposase binding sites placed in a direct repeat (IR/DR) orientation separated by DNA segments of variable sequence^1^. To date, little biochemical data is available for the *SB* transposon and structural information is limited to the N-terminal HTH-motif of the transposase DBD^24^. To facilitate *SB*'s applications in genetic engineering, several hyperactive transposase variants have been developed^2^,^25^,^26^,^27^, providing up to 100-fold increased efficiencies. However, the activity of the transposase is cell type dependent^28^ and several applications, including gene transfer into medically relevant primary human cells, would greatly benefit from further enhanced transposase variants. However, rational engineering of novel SB variants is currently hampered by the lack of specific mechanistic and structural data, particularly for the transposase catalytic domain. To overcome this limitation, here we solve the first crystal structure of the SB catalytic domain and, based on this, generate a model of the SB transpososome containing transposon end as well as target DNA. The acquired structural insights enable us to rationalize previous hyperactive mutations on the transposase as well as design novel defective and hyperactive SB variants. Our work provides novel insights into SB's mechanism and demonstrates how structure-based design can help generate further designer transposases.

## Results

### The structure of the SB100X catalytic domain

**Figure 1:**
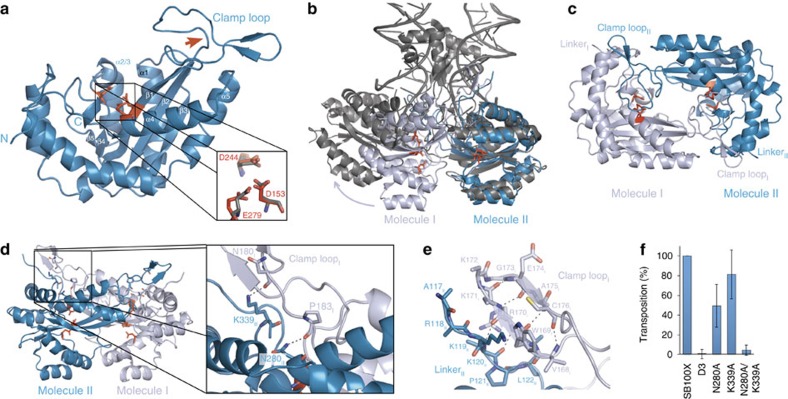
The structure of the SB100X transposase catalytic domain. (**a**) The SB100X catalytic domain (blue) assumes an RNaseH-fold with all catalytic residues (red) assembled in the active site. Conserved α-helices (α) and β-strands (β) are indicated. Insert: superposition of active-site residues in SB100X and Mos1 (grey, PDB 3HOS)^6^. (**b**) The SB100X dimer (dark and light blue) observed in our crystal structure (molecules I and II) superposed onto the Mos1 PEC structure (grey). Arrow illustrates the rearrangement (∼50° swing to left with ∼20° rotation backwards) that can bring Molecule I into the PEC conformation. (**c**) Top view of the SB100X catalytic domain dimer highlights the role of the clamp loops in the interaction. (**d**) Residues N280 and K339 of the RNaseH core form sequence-specific interactions (hydrogen bonds indicated with dashed lines) with the clamp loops of the partner molecules, anchoring them to cover the active sites. Insert: close-up of the dimer contacts mediated by N280 and K339, respectively. (**e**) β-stranded interactions between the backbones of the clamp loop and the inter-domain linker. (**f**) Transposition assay for N280A and K339A mutants in HeLa cells. D3 indicates the negative control using a catalytically inactive transposase mutant, E279D. Error bars represent the means±s.e.m. of three independent experiments. Statistical analysis was performed by a one-sample *t*-test and *P* values are as follows: N280A, 0.061; K339A, 0.34; and N280A/K339A, 1.7 × 10^−4^.

To provide structural insights into *SB* transposition, we crystallized the catalytic domain (aa 114–340; including most of the flexible inter-domain linker that spans aa 110–127) of the current most active SB transposase variant^2^, SB100X, and solved its structure at 1.4 Å resolution (Fig. 1a, Supplementary Fig. 1 and Table 1). The core of the structure reveals a canonical RNaseH-fold, consisting of a central five-stranded β-sheet (β1–β5) surrounded by five α-helices (α1–α5). The catalytic residues (D153, D244 and E279, red in Fig. 1a) are assembled in close proximity establishing an active site conformation similar to the one observed in the crystal structure of the homologous Mos1 transposase PEC (grey in Figs 1a,b)^7^.

In addition to the core RNaseH domain, most of the inter-domain linker (aa 117–127) and also the flexible clamp loop (aa 159–190), which is inserted between β1 and β2 of the RNaseH-fold^7^ and includes part of the glycine-rich strip (aa 183–190)^1^, are visible in the structure. While the RNaseH core superposes closely with that of Mos1 in the PEC (r.m.s.d. 1.97 Å for Cα atoms, Fig. 1b) the linker and the clamp loop assume different conformations (Supplementary Fig. 2). In the Mos1 PEC, the clamp loop is extended and interacts with the similarly extended linker and both tnp ends across the dimer interface, playing an important role in PEC assembly^7^. In turn, in our SB100X catalytic domain crystals, the clamp loop is curved (Fig. 1a and Supplementary Fig. 2b), mostly pivoting on three consecutive G-s (aa 188–190, marked with red arrow in Fig. 1a) in the glycine-rich strip, and contributes to an extended protein–protein interface (2,350 Å^2^ surface area; ΔG=−27.2 kcal mol^−1^)^29^ between symmetry related molecules in neighbouring asymmetric units (Fig. 1b,c and Supplementary Fig. 3). This interface brings two catalytic domains into close proximity with their active sites facing each other in an arrangement that resembles the one in the Mos1 PEC^7^ (Fig. 1b), but is more compact and features several additional tight sequence-specific interactions (Fig. 1d,e and Supplementary Fig. 3g,h). The clamp loops of both protomers form reciprocal interactions with the RNaseH core of the partner molecule, covering the active sites (Fig. 1c,d and Supplementary Fig. 2b). In addition, the tip of the clamp loop contains two short antiparallel β-strands (aa 169–171 and 174–176), which form a β-hairpin and interact with the main chain of the inter-domain linker (aa 119–122) of the partner molecule (Fig. 1e and Supplementary Fig. 3g). This contact resembles the β-stranded clamp loop—linker interaction observed in the Mos1 PEC, but involves a different part of the linker, which is structurally equivalent to the regulatory WVPHEL motif of mariner transposases^15^,^16^,^30^.

To investigate the functional relevance of the observed interface, we mutated amino acids N280 and K339 on the RNaseH core (Fig. 1d) and assessed the effects in transposition assays in HeLa cells (Fig. 1f and Supplementary Fig. 4). These amino acids were selected for mutagenesis, because they are critical to the newly observed interface, but localize distant from the intermolecular interface in the PEC and have not been attributed a functional role before. We find that simultaneous mutation of both residues strongly reduces transposition (*P*=1.7 × 10^−4^; *t*-test), indicating that their combined interactions are required for efficient transposition (Fig. 1f). However, as the observed arrangement of the catalytic domains is too compact to allow DNA to access the active sites (Fig. 1b), it does not appear compatible with PEC assembly. Instead, it might mediate the formation of a pre-catalytic dimer that must still undergo conformational rearrangement upon PEC formation (illustrated by the arrow in Fig. 1b).

### The SB transposase/tnp end/tDNA complex model

**Figure 2:**
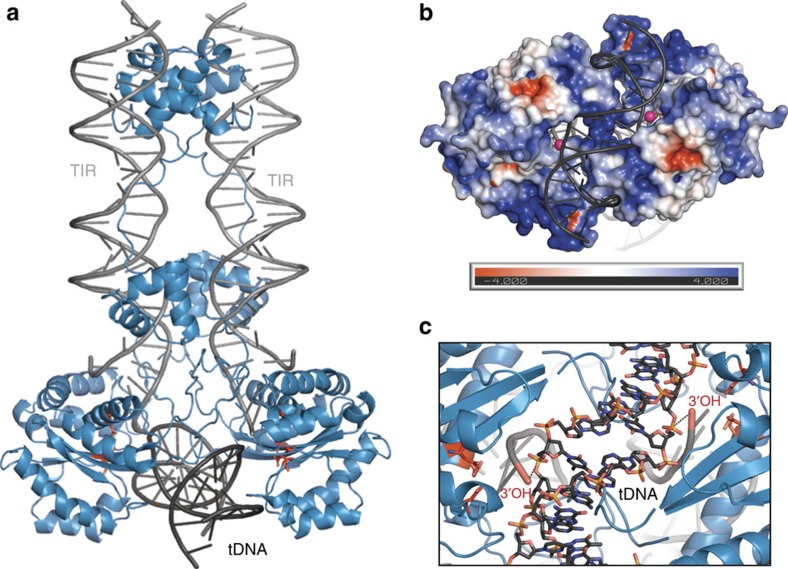
The SB TCC model. (**a**) Cartoon representation of the model: SB100X dimer (blue), tnp ends (TIRs, grey) and bent tDNA substrate (tDNA, dark grey). (**b**) Surface representation of the tDNA-binding groove coloured by electrostatic potential calculated using APBS^48^ at 150 mM salt. The kinked tDNA (grey cartoon) fits well in the positively charged groove. Pink balls represent the 3′OH groups of the tnp TS. (**c**) The tDNA (stick representation with atomic colouring) scissile phosphates approach the active sites of the TCC.

To evaluate and exploit the predictive value of our SB100X structure for mechanistic studies and protein engineering, we next aimed to place it into the functionally relevant context by modelling the SB tDNA capture complex (TCC), which contains the full-length transposase in complex with tnp end and target DNA (Fig. 2a). Since an intact TCC structure is so far not available for any *Tc1/mariner* transposon, we used the Mos1 PEC structure^7^ to model the SB100X PEC (combining a homology model of the DNA-binding domains with the catalytic domain coordinates determined here), and docked a bent tDNA substrate derived from the prototype foamy virus (PFV) intasome structure^23^ into the positively charged cleft formed at the base of the catalytic domains (Fig. 2b).

We used a bent tDNA for our modelling, as previous biochemical and structural data have indicated that *Tc1/mariners* require significant target bending for efficient integration^16^,^17^,^18^,^21^, a feature that seems to be broadly conserved among DDE enzymes^22^,^23^,^31^. In accordance, the tDNA-binding groove in the SB100X TCC model has an inward arc with the active sites situated at the bottom (Fig. 2b) and cannot fit a straight B-form DNA without major clashes. In turn, the kinked PFV tDNA fits well in the cleft with its sugar-phosphate backbones delving into the active sites. In the resulting TCC model the two tnp ends are arranged in parallel, with the 3′OH groups of the TS approaching the phosphate groups flanking the central TA on opposite strands of the tDNA (P-O distances: 4.8 and 5.3 Å, Fig. 2c).

### The SB TCC model rationalizes previous hyperactive mutations

**Figure 3:**
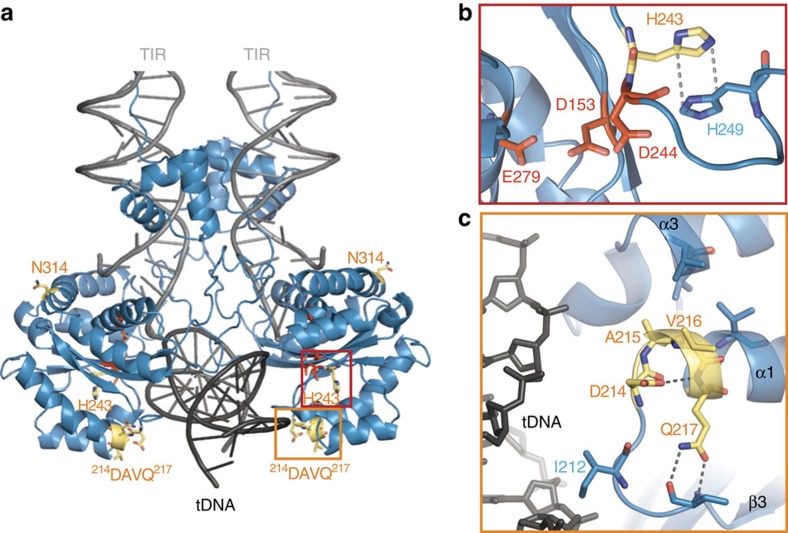
SB100X structure explains previous hyperactive mutations. (**a**) The TCC model with catalytic domain residues mutated for the generation of SB100X shown in yellow. The localization of the interactions shown in (**b**,**c**) is indicated by red and orange rectangles, respectively. (**b**) H243 stabilizes the catalytic core (red) via π-stacking with H249 (indicated with dashed lines). (**c**) Residues ^214^DAVQ^217^ stabilize a flexible loop (including I212) and tether it to the tDNA-binding surface. Dashed lines indicate hydrogen bonds.

Using the TCC model, we first aimed to rationalize previous mutations within the SB100X catalytic domain that contributed to generation of the hyperactive SB100X transposase^2^. These comprise RKEN214–217DAVQ (ref. 27), M243H, and T314N (Fig. 3). We find that N314 is exposed on the TCC surface, where the mutation can improve transposase solubility, which has been shown to be a limiting factor for transposition^2^. H243 is situated next to the second catalytic residue D244 (Fig. 3b) and establishes a parallel-displaced π-stack with H249, structuring the 243–251 loop and helping to position D244 in the active site. Finally, residues 214–217 are located on the beginning of helix α1, immediately following the loop connecting β3 to α1 (aa 209–214), which forms part of the target-binding groove (Fig. 3c). D214 and Q217 side chains form hydrogen bonds with main chain atoms in β3 and α1, intimately connecting these elements and stabilizing the connecting loop conformation. Simultaneously, A215 and V216 form hydrophobic interactions with residues in the neighbouring α3 helix, positioning the loop on the target-binding surface. Thus, the 214–217 mutations have likely helped shape and ideally position the β3–α1 linker to interact with the tDNA at the rim of the binding groove.

In addition, the structure offers a rationale for other previously reported hyperactive mutations that are not present in SB100X. For example, the ALHKID205–210KLVRIE mutations^27^ probably have a similar effect on the β3–α1 loop as the RKEN214–217DAVQ mutation; the M243Q mutation in SB11 (ref. 25) likely stabilizes the active site the same way as M243H; while surface mutations (for example, V253H, V255R^25^, T295N^2^ and D260K^26^) improve transposase solubility.

### Structure-based design of tDNA interaction mutants

**Figure 4:**
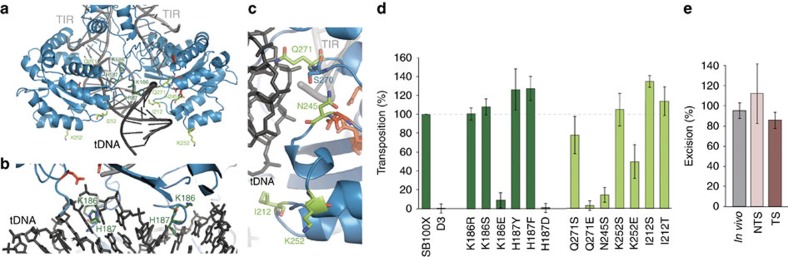
Structure-based mutagenesis maps tDNA binding and generates novel hyperactive transposases. (**a**) TCC model with predicted tDNA-binding residues selected for mutagenesis. (**b**,**c**) Zoom-ups on residues predicted to be involved in tDNA recognition or bending (dark green, **b**) and residues lining the target-binding groove (light green, **c**). (**d**) Transposition assay for the mutants in HeLa cells. *P* values (based on one-sample *t*-test): K186R, 0.93; K186S, 0.22; K186E, 0.0019; H187Y, 0.069; H187D, 6.9 × 10^−6^; Q271S, 0.21; Q271E, 7.5 × 10^−5^; N245S, 0.0022; K252S, 0.68; K252E, 0.042; I212S, 0.0047; and I212T, 0.27. D3 indicates the negative control with the inactive E279D transposase. (**e**) Excision assays with the hyperactive I212S transposase *in vivo* and *in vitro* (monitoring cleavage on both DNA strands: NTS, TS). Results are normalized to SB100X. All error bars represent the s.e.m. for three independent experiments.

Next, we used our TCC model to map transposase residues that may be involved in tDNA-binding (green in Fig. 4a–c), and tested the effect of their mutations in transposition assays (Fig. 4d and Supplementary Fig. 4). Overall, we selected two sets of amino acids for mutation: (i) amino acids located near the integration site that may be involved in TA recognition and/or tDNA bending (K186 and H187; Fig. 4b, dark green); and (ii) residues that are likely involved in unspecific tDNA binding on the flanks of the integration site (I212, N245, K252 and Q271; Fig. 4c, light green); then, we designed mutants to either abolish the predicted function of these residues or to generate a hyperactive phenotype. In the first set, K186 and H187 localize to the end of the clamp loop, which delves into the major groove of the tDNA substrate in our TCC model and might contribute to broadening the groove and/or kinking the tDNA substrate via unstacking bases at the integration site (Fig. 4b). In Mos1, R186 was proposed to be essential for target recognition and its alanine mutation abolished integration^7^. Interestingly, the equivalent K186S mutation in SB100X does not affect transposition (Fig. 4d), indicating that the K186 side chain is not directly responsible for integration site recognition in SB, while the K186E mutation abolishes transposition (*P*=10^−4^; *t*-test) confirming that proximity of this protein segment to DNA is essential. In addition, we find that mutations of H187 also affect transposition: introduction of an aspartate (H187D) abolishes transposition (*P*<10^−5^; *t*-test), while aromatic residues (H187F/Y)—that can function well in tDNA bending—support activity and even exhibit hyperactive phenotypes (*P*=0.069 and 0.19 based on *t*-test for F and Y, respectively) relative to SB100X. This suggests that in SB, H187 (rather than K186) might fulfil a function in target recognition by side chain specific interactions with tDNA bases at the integration site in a way similar to R186 in Mos1.

Our second set of mutants (I212, N245, K252 and Q271) localizes to the surface of the catalytic domain within the tDNA-binding groove (Fig. 4c,d). For Q271, the TCC model suggests that its side chain directly contacts the tDNA backbone and helps target binding. In agreement, both Q271S and Q271E mutants exhibit reduced transposition (*P*=0.21 and <10^−4^, respectively; *t*-test). N245 forms a hydrogen bond with S270 and helps position the loop that contains Q271. Consistently, the N245S mutant is also practically inactive (*P*=0.0022; *t*-test). In turn, K252 is situated on the verge of the tDNA-binding groove fairly distant from the tDNA position predicted by our model, so that its positive charge might contribute to the overall DNA affinity, but is not directly involved in tDNA recognition. In agreement, the K252S mutation has no effect and only the K252E mutation reduces transposition (*P*=0.042; *t*-test). Finally, I212 is located on the β3–α1 loop next to the DAVQ stretch (Fig. 3c) near the tDNA. While its position is ideal for tDNA binding, its hydrophobic side chain prohibits such contacts. Thus, to allow for direct DNA contact and increase tDNA affinity, we mutated I212 to short hydrophilic residues. We find that the I212S mutant transposes 30% more efficiently than SB100X (*P*=0.0047; *t*-test). Since the transposon excision activity of I212S is unaltered *in vivo* and *in vitro* (Fig. 4e and Supplementary Fig. 5), the observed hyperactive phenotype is likely due to increased tDNA affinity as predicted from the structure.

## Discussion

The most surprising feature of the SB100X catalytic domain structure concerns the clamp loop, which is inserted within the RNaseH-fold of Tc1/mariner transposases. Unlike in previous structures of Mos1 and SETMAR/Metnase^7^,^32^,^33^, in SB100X the clamp-loop assumes an unusually bent conformation and creates a large symmetric intermolecular interface connecting two catalytic domains. Although this interaction might be provoked by protein truncation or crystal packing, its extended size and low ΔG argue for a significant functional relevance^29^. In accordance, disrupting the interface by mutagenesis diminishes transposition in cell-based transposition assays (Fig. 1d,f).

Concerning a potential functional role of the interaction, it is unlikely to represent a PEC assembly, because the arrangement of the catalytic domains is too compact to allow DNA to access the active sites (Fig. 1b). However, accumulating biochemical data indicate that Tc1/mariner transposases form multiple conformationally distinct dimeric states throughout their transposition pathway^7^,^8^,^13^,^14^,^15^, and the dimeric assembly observed here could represent one of the proposed DNA-free or single end-bound pre-catalytic states, which must still undergo conformational rearrangement upon PEC formation (see arrow in Fig. 1b). The auto-inhibited dimer arrangement proposed by our SB100X structure would offer two distinct benefits for the *Tc1/mariner* transposition pathway. First, it could facilitate recruitment of a sufficient number of transposases to the distantly located transposon ends in the genome, thereby facilitating PEC assembly without introducing futile DNA breaks. Second, as it closely resembles the PEC, it would alleviate the need for major conformational changes upon PEC assembly. The conformational rearrangement required for activation would only entail a simple rotation of one catalytic domain (Fig. 1b, ∼50° swing to with a ∼20° backwards rotation) and repositioning of the flexible clamp loop, which can likely occur easily without requiring significant energy. In addition, for SB in particular such a pre-catalytic dimer might also help facilitate transposase recruitment to the tnp ends via the second transposase binding site in the TIR that has been shown to be required for efficient transposition but is never cleaved. Thus, the assembly observed here could also be a unique feature of SB contributing to its exceptional efficiency.

The proposal that the interaction observed between the SB100X catalytic domains in the crystals represents a functionally relevant transposase dimer is further supported by the fact that the β-strand interaction observed between the clamp loops and the linkers of the two transposase subunits resembles the inter-subunit interactions in the Mos1 PEC, which play an important role in PEC assembly and coordination^7^. However, the molecular details of the interactions are different: in the Mos1 PEC they involve only one β-strand of the clamp loop (aa 168–172) and the first β-strand of the linker (aa 113–116), while in the SB100X structure the interaction extends to the second β-strand of the clamp loop (aa 173–176) and involves a different part of the linker (aa 119–122) (Fig. 1e). Remarkably, residues 119–122 belong to a highly conserved sequence stretch (KKPLLS) in *Tc1*-like elements that is structurally equivalent to the WVPHEL motif of mariner transposases, which has been shown to play a critical role in orchestrating cleavage events within the transpososome^15^,^16^,^30^. This suggests that different *Tc1/mariner* transpososomes might use similar molecular strategies involving the same flexible protein segments (that is, the clamp loop and the linker) to exhibit multifaceted regulatory functions during their transposition pathway and the interaction observed here might represent an important regulatory state.

Molecular modelling and cell-based mutagenesis data presented here indicate that the SB100X transposase uses a bent tDNA for integration. This is in good agreement with previous work on various *Tc1/mariner* elements^9^,^17^,^18^,^19^,^20^,^21^, implying that tDNA bending is a widespread feature within this superfamily. In addition, strand transfer complex structures of more distantly related DDE enzymes, the Mu phage transposase (MuA) and the PFV integrase, indicated that tDNA bending is conserved more broadly, likely serving to drive transposition forward by rendering strand transfer irreversible via straining the DNA such that it snaps away from the active sites after integration^22^,^23^. However, available crystal structures revealed a remarkable diversity of transpososome architectures and the degree of tDNA bending used by individual systems seems to differ greatly. For instance, the Tn5 transpososome performs integration with a 9-bp stagger from the two opposite sides requiring little, if any, tDNA bending^34^. In turn, PFV introduces a ∼90° bend in the target with a single kink in the middle that is attacked by the two ends of the viral DNA from the same side with a 4-bp stagger. Finally, MuA introduces two kinks and a significant unwinding to bend the tDNA sharply (∼140°) for integration with a 5-bp stagger.

In SB100X, the positively charged groove created by the transposase catalytic domains in the TCC model accommodates a ∼90° bent tDNA (from the PFV intasome) well (Fig. 2b), and mutagenesis data supports this docking (Fig. 4). Although this model approximates how tDNA might be bound in general, we suspect that further kinks or unwinding will occur around the target TA. Additional bending, perhaps similar to the one observed in the Mu transpososome (P-P distance 24.5 Å on a 2 bp step flanking the cleavage site)^22^, would help increase the distance between the scissile phosphates (only 19.1 Å apart in the PFV tDNA) improving their positioning in the active sites (that are 24.8 Å apart, measured on the 3′OH groups of the TS, Fig. 2c). This idea is consistent with the hyperactive phenotypes of the H187 F/Y mutants, which likely help improve tDNA bending.

One of the unique features of the SB transposase compared with better-characterized relatives like Mos1 and HsMar1 is the Tc1 subfamily specific glycine-rich strip at the base of the clamp loop. The structural data presented here proposes two distinct functions for this motif. Firstly, owing to its flexibility, it might be critical to the formation of a pre-catalytic transposase dimer, thereby helping to control the pathway of transposition. Secondly, as the glycine-rich strip is located close to the TA target site in the TCC, its ‘naked' peptide bonds could insert in the major groove, helping to bend the tDNA. Although supported by the data presented here, these proposals have to be validated through experimental analysis of the complete SB TCC structure.

The *SB* transposon is a versatile genetic tool offering widespread applications for genetic manipulations in research and medicine. Although the SB transposase has been mutagenized extensively^2^,^25^,^26^,^27^, rational design of improved variants has not been possible so far in lack of structural and mechanistic data. The structure of the SB100X catalytic domain determined here opens up avenues for structure-based engineering of designer SB variants. For example, it might be possible to further enhance transposase activity or increase/modulate target site specificity. Hyperactive variants could further enhance gene transfer rates greatly facilitating gene therapy applications. Increasing target specificity would additionally improve the fidelity of this genetic tool, providing an added benefit in medical applications. Alternatively, it might be possible to create variants with uncoupled excision and integration activities. The utility of such tools e.g., in stem cell engineering has been already demonstrated for *piggyBac*^35^,^36^, but they might also help increase the specificity of target site selective SB-variants^37^.

In summary, the work presented here describes the atomic resolution structure of the SB100X transposase catalytic domain and shows that it provides a useful resource for transposase engineering. Using the structure we create a model of the complete transpososome with target DNA bound and map transposase residues involved in tDNA binding. By mutational analysis we validate the transpososome architecture and generate two variants with efficiencies higher than SB100X, demonstrating that structure-based design can enable the creation of further hyperactive or designer SB transposases for genetic engineering.

## Methods

### Protein production and purification

The full-length SB100X transposase, its catalytic domain, and the full-length I212S mutant were expressed recombinantly and purified for *in vitro* biochemical assays and crystallization. The full-length *SB100X* gene^2^ was first cloned into BspHI (5′-GATCTCATGATGGGAAAATCAAAAGAAATCAGCC-3′) and XhoI (5′-GATCCTCGAGCTAGTATTTGGTAGCATTGCC-3′) sites of the pETM22 (PepCore, EMBL Heidelberg) vector and the I212S mutation was then introduced via site-directed mutagenesis. The SB100X catalytic domain construct (aa 114–340) was generated via restriction-free cloning into pETM22 (fw: 5′-GGAAGTTCTGTTCCAGGGGCCCAGCGGCCACTCAGCAAGG-3′ and rev: 5′-CGGATCCGGTACCTCATTAGTCCATCTAGTATTTGGTAGCATTGCCTTTA-3′). All proteins were expressed with an N-terminal ThioredoxinA (TrxA)-6xHis-tag in *Escherichia coli* RosettaII(DE3)* cells (Novagen) in LB medium at 16 °C for 18 h. The fusion proteins were purified on a Ni^2+^-Sepharose column (in 1 × PBS, 1 M NaCl, 5% (v/v) Glycerol, 20 mM Imidazole, 0.2 mM TCEP) following the manufacturer's instructions (GE Healthcare). The eluate was incubated with PreScission protease (PepCore, EMBL Heidelberg) for 18 h at 4 °C to remove TrxA-6xHis. The cleaved tag was removed by Ni-affinity purification and the protein was further purified by size-exclusion chromatography on a Superdex 200 16/60 column (GE Healthcare) in 1 × PBS, 1 M NaCl, 5% (v/v) Glycerol, 1 mM MgCl_2_ and 0.2 mM TCEP.

### DNA oligonucleotides

DNA oligonucleotides were purchased PAGE-purified from Integrated DNA Technologies. The 52 nt dsDNA substrate used for *in vitro* cleavage assays was generated via 5′-labelling of individual oligonucleotides (5′-GAGCTCGGTACCCTATACAGTTGAAGTCGGAAGTTTACATACACTTAAGTTG-3′ or 5′-CAACTTAAGTGTATGTAAACTTCCGACTTCAACTGTATAGGGTACCGAGCTC-3′) with ^32^P before annealing.

### Crystallization and structure determination

Two microlitre (μl) of the purified SB100X catalytic domain (aa 114–340) at 19 mg ml^−1^ (in 20 mM Hepes (pH 7.2), 50 mM NaCl, 2% (v/v) Glycerol, 10 mM MgCl_2_, 0.2 mM TCEP) were mixed with 2 μl of precipitant solution (0.1 M NaCitrate pH 4.8, 3.2 M (NH_4_)_2_SO_4_). Crystals grew within 5 days at 4 °C in hanging drop vapour diffusion plates and were flash frozen in liquid nitrogen after addition of 5% glycerol as cryo-protectant. X-ray diffraction data were collected at 100 K at 0.9763 Å wavelength on the PETRAIII beam line P14 (EMBL/DESY, Hamburg, Germany). Diffraction images were processed and scaled using XDS^38^ and XSCALE^39^. The structure was solved by molecular replacement in Phaser^40^ using the catalytic domain of Mos1 (from PDB ID 3HOS)^7^ as search model. The solution was refined through iterative cycles of model building in COOT^41^ and refinement in Phenix^42^ and was validated using MolProbity^43^ (Table 1). The final structure has very good geometry with 98% of protein residues in favoured Ramachandran regions and no outliers.

### Modelling

For modelling the SB TCC structure, a homology model was generated for the full-length SB100X transposase in I-TASSER^44^ based on the coordinates of the Mos1 PEC^7^ (PDB ID 3HOS) using the coordinates of the here determined catalytic domain structure as restraint. Since I-TASSER generated small alterations in the catalytic domain coordinates relative to the actual structure, the catalytic domain coordinates were then replaced with the ones from the structure except for the clamp loop and the linker (the conformation of which is expected to change upon PEC formation), where the model coordinates were used. The arrangement of two transposase monomers and the positioning of the tnp end DNA in the complex was derived from superposition with the Mos1 PEC^7^. The bent tDNA substrate was taken from the PFV intasome structure (PDB 3OS1)^23^ and manually docked into the tDNA-binding groove generated by the two SB catalytic domains. For docking, the distance between the 3′OH of the tnp ends and the scissile phosphates in the tDNA were restrained to 5 Å. Manual modelling operations were performed in Chimera^45^ and Pymol^46^, and molecular graphics were generated in Pymol.

### *In vivo* transposition assays

Overall *SB* transposition activity was quantified in HeLa cells (ATCC Cell Lines) using the protocol described in^1^ that was fine-tuned to obtain single-copy insertions. In short, transposon donor plasmids containing a resistance-encoding transposon (pT2Bpuro) were co-transfected with plasmids encoding the full-length SB100X or mutated SB100X transposase variants (pCMV(CAT)T7-SB100X). The catalytically inactive mutant, E279D (referred to as D3)^2^ was used as negative control. 48 h after transfection, cells were trypsinized and selected for transposon integration using 3 μg ml^−1^ puromycin (InvivoGen). Surviving colonies were fixed, stained with methylene blue and counted. At least three independent experiments were performed for every mutant. All data points were averaged and standard errors were calculated using a James–Stein-shrinkage estimator of the corresponding variances^47^. To evaluate statistical significance, two-tailed one-sample *t-*test was performed.

Expression of mutant proteins was tested via western blotting 48 h after transfection. Cells were lysed and proteins were extracted via sonication at 4 °C. Total protein was quantified using a BCA Protein Assay Kit (Pierce) and 10 μg per lane were loaded onto 10% polyacrylamide gels and subjected to SDS–polyacrylamide gel electrophoresis. Gels were transferred to nitrocellulose membrane (Hybond ECL, Amersham Bioscience, Little Chalfont, UK) and immunoblotting was performed according to standard procedures. Bands were detected with anti-SB (R&D Systems; Catalog Number AF2798; dilution 1:5,000) or anti-Vinculin (Abcam; Catalog Number ab18058; dilution 1:3,000) antibodies, visualized with chemiluminescent reagents (ECL Prime Western Blotting Detection Kit, Amersham Bioscience) and imaged on films (Hyperfilm ECL High performance, Amersham Bioscience). Correct localization of the mutant proteins was confirmed by immunofluorescence staining. Cells were fixed with 4% PFA in PBS 48 h post transfection, incubated with anti-SB (R&D Systems) primary antibody and stained with Alexa Fluor 488-labelled secondary antibody (Life Technologies) and DAPI (Invitrogen). Cells were examined on a Nikon Eclipse Ti-S inverted microscope (40x objectives).

### *In vivo* excision assays

*SB*'s excision activity was quantified using a fluorescence-activated cell sorting (FACS)-based excision assay in HeLa cells (ATCC Cell Lines; Supplementary Fig. 5c). To this aim, plasmids expressing SB100X (above) were transfected together with a tnp donor plasmid (pCMV(CAT)-GFP//T2Neo). The transposon donor contained a green fluorescent protein (GFP) open reading frame disrupted by a *SB* tnp insertion carrying a neomycin (neo) resistance gene. Precise excision of the transposon restores the GFP open reading frame leading to fluorescent cells that can be detected by FACS analysis to quantify excision frequencies. A GFP-expressing *SB* tnp plasmid (pT2.CAGGS.AmGFP) was used as control. Three days post-transfection cells were trypsinized, washed with PBS, fixed with 1% PFA in PBS and FACS analysed with a BD LSR II flow cytometer (BD Biosciences). Data were evaluated with FCS Express 4 Flow Cytometry (De Novo Software). Excision frequencies determined for mutated SB100X variants were normalized against the values measured for the SB100X protein.

### *In vitro* cleavage assays

SB100X or the I212S mutant were mixed with LO52 (20 nM) at a molar ratio of 10:1 in 20 mM Hepes (pH 7.2), 150 mM NaCl, 10 mM MgCl_2_ and 1 mM DTT. Reactions were incubated at 25 °C for 20 h and terminated by Proteinase K (New England Biolabs) treatment according to the manufacturer's instructions. Reaction products were ethanol-precipitated, re-suspended in 2 × formamide loading dye and analysed by gel electrophoresis on TBE-urea 12% acrylamide/bis-acrylamide (19:1) gels. Gels were imaged on a FLA 7000 phosphoimager (Fuji) and quantified in the Fujifilm Multi Gauge software package.

## Additional information

**Accession codes:** Coordinates and structure factors have been deposited in the Protein Data Bank under accession code 5CR4.

**How to cite this article:** Voigt, F. *et al.* Sleeping beauty transposase structure allows rational design of hyperactive variants for genetic engineering. *Nat. Commun.* 7:11126 doi: 10.1038/ncomms11126 (2016).

## Supplementary Material

Supplementary Information Supplementary Figures 1-5 and Supplementary References.

## Figures and Tables

**Table 1:** X-ray crystallographic data collection and refinement statistics.

*Data collection*	
Space group	I 2_1_ 2_1_ 2_1_
Cell dimensions	
*a, b, c* (Å)	84.24, 113.98, 144.94
*α*, *β*, *γ* (°)	90, 90, 90
Resolution (Å)	50–1.4 (1.50–1.40)*
*R*_sym_	0.081 (1.707)
*I*/σ*I*	17.9 (2.0)
Completeness (%)	100.0 (100.0)
Redundancy	11.2 (11.2)
No. unique reflections	136,613 (25,318)
CC(1/2)	1 (0.694)
	
*Refinement*	
Resolution (Å)	50–1.4
No. reflections, all/free set	136,607/6,830
*R*_work_*/R*_free_	0.171/0.195
No. atoms	
Protein	3,782
Ligand/ion	219
Water	721
*B*-factors	25.40
Protein	22.40
Ligand/ion	39.60
Water	36.80
R.m.s deviations	
Bond lengths (Å)	0.020
Bond angles (°)	1.91

^*^Highest resolution shell is shown in parenthesis.
