# Supplementary material for: Sleeping Beauty transposase structure allows rational design of hyperactive variants for genetic engineering
Source: Nat Commun. 2016 Mar 30;7:11126. doi: 10.1038/ncomms11126 (PMC4820933; doi:10.1038/ncomms11126)
Supplement: Supplementary Information — Supplementary Figures 1-5 and Supplementary References. [file ncomms11126-s1.pdf]

a

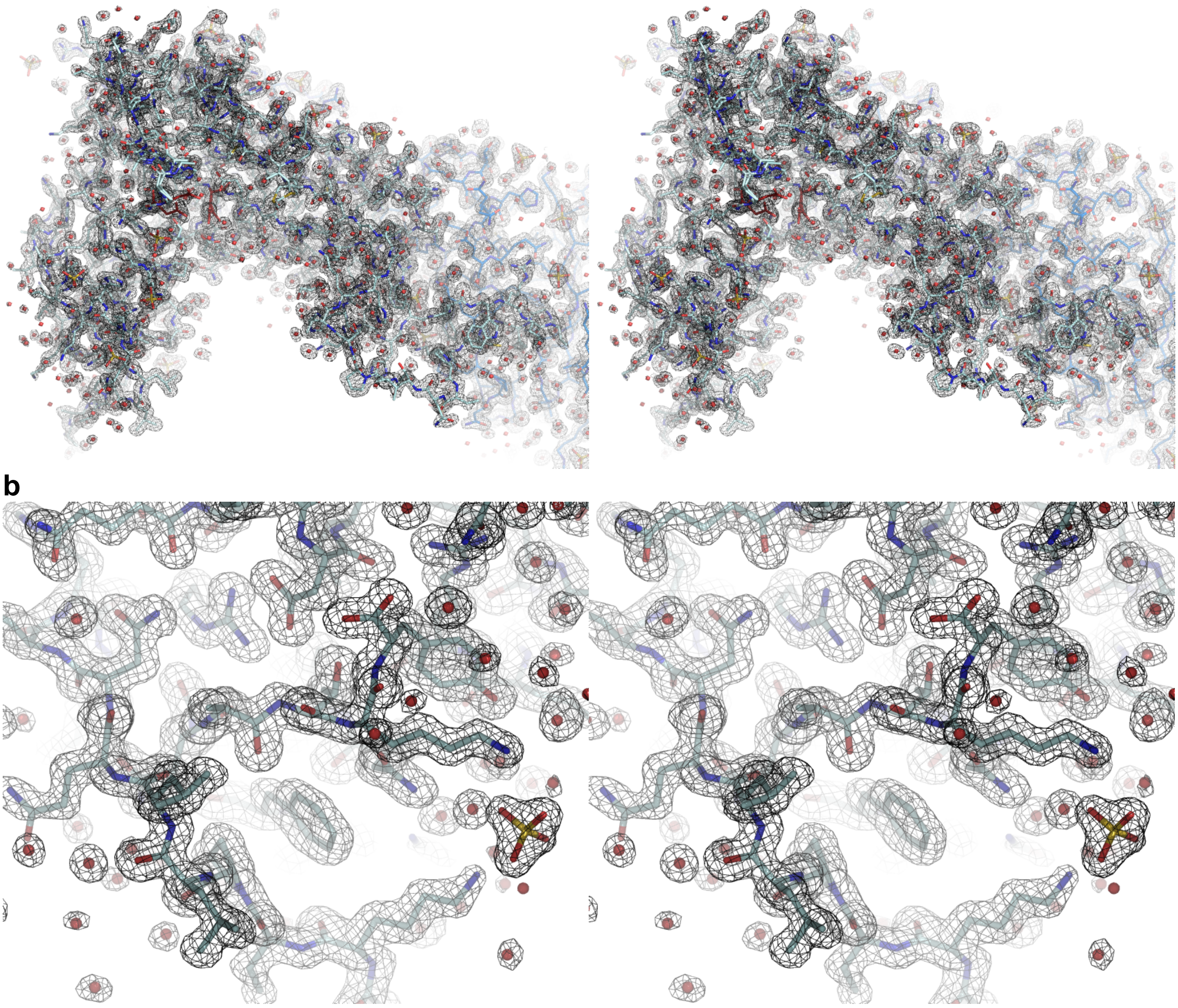

Supplementary Figure 1. Stereo images of portions of the 2Fo-Fc electron density maps contoured at 1.5 sigma. (**a**) Wall-eyed stereo image showing the bias minimized 2Fo-Fc composite omit electron density map (black mesh) for the entire SB100X catalytic domain structure (cyan). Part of the second molecule (blue) present in the asymmetric unit is visible in the back of the image. Active site residues are highlighted in red sticks and water molecules are shown as red balls. (b) Close up showing a representative portion of the map.

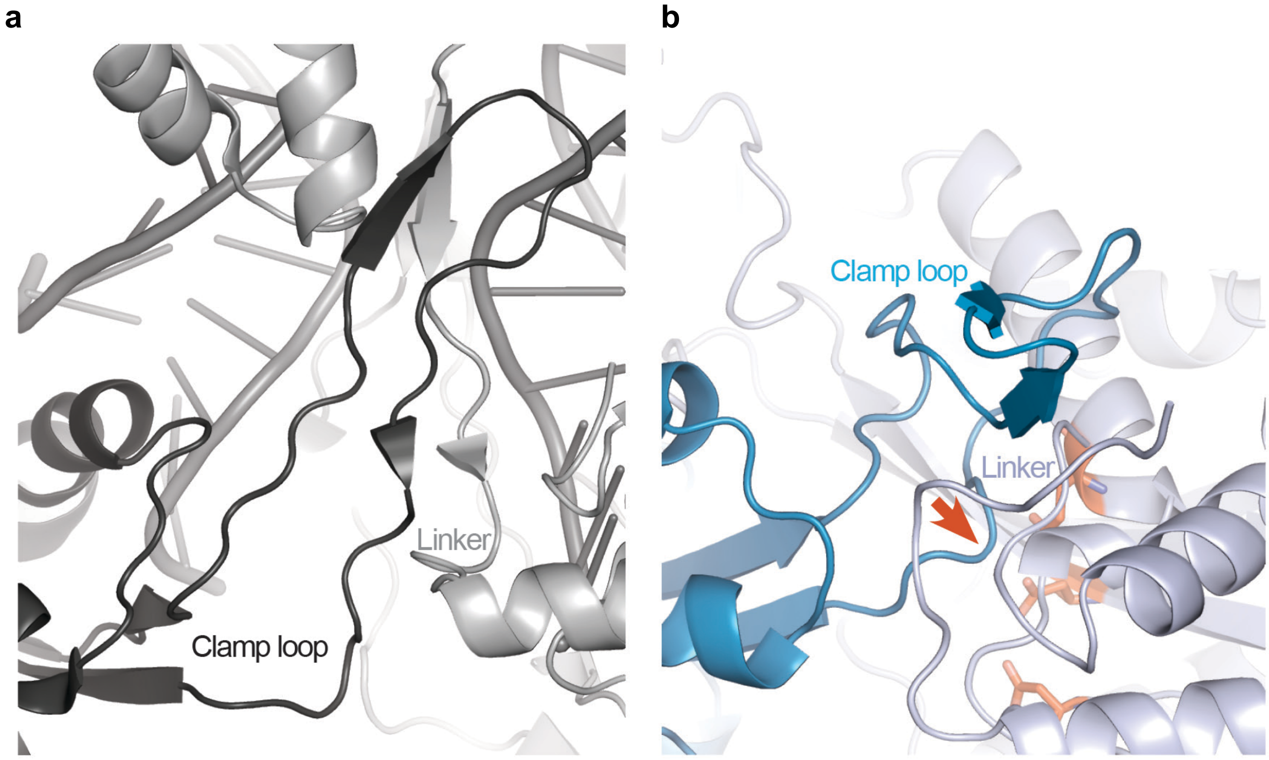

Supplementary Figure 2. Side-by-side comparison of the clamp loop conformation in the Mos1 PEC[1] (a) and in the SB100X catalytic domain structures (b). Mos1 dimer in the PEC is shown in two shades of gray and the SB dimer observed in our crystal structure is shown in dark and light blue. The clamp loops and linkers are marked, and the position of G188-G190 in the glycine-rich strip is indicated with red arrow.

a

ID	Mol. I	Mol.II	Symmetry operation	Interface area [Å^2]	ΔG [kcal/mol]
1	A	A	x, -y, -z-$^{1}/_{2}$	2375.1	-27.2
2	B	B	-x, -y+$^{1}/_{2}$, z	2314.6	-22.5
3	B	A	x, y, z	741.1	-1.5
4	B	B	-x+$^{1}/_{2}$, y, -z	736.0	-6.8
5	A	A	-x-1, -y+$^{1}/_{2}$, z	677.1	0.3

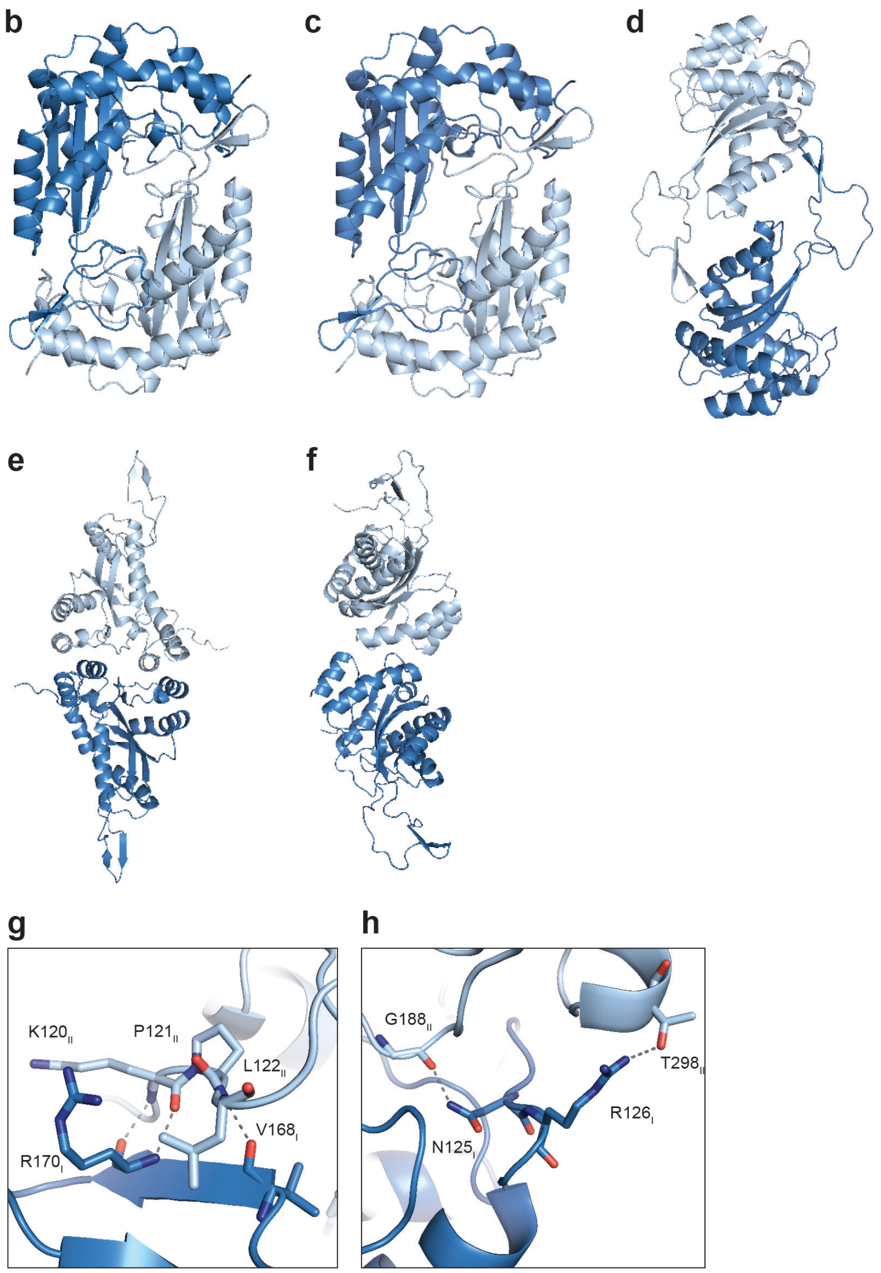

Supplementary Figure 3. Intermolecular interactions in the SB100X catalytic domain crystals. (**a**) PISA[2] analysis of the five most extended interfaces observed in the crystal structure. (**b-f**) Cartoon representations of the interfaces 1-5. Interacting molecules are colored in two shades of blue. Interface 1 (**b**) and 2 (**c**) involve Molecules A and Molecules B, respectively, from neighboring asymmetric units, and are practically identical. They distantly resemble the molecular arrangement in the PEC. Interface 3 (**d**) connects the two molecules in the same asymmetric unit. Interface 4 (**e**) resembles the interface described for the transposase domain of Metnase (PDB: 3K9J)[3]. (**f**) Interface 5 is unlikely to be physiologically relevant because it has a positive ΔG. (**g, h**) Molecular details of the interactions on the dimer interface 1. Interacting residues are shown in stick representation. Hydrogen bonds are indicated with dashed lines. See also Fig.1d,e for additional contacts. (**g**) Another view of the hydrogen bond network between the backbones of the clamp loop (molecule I, dark blue) and the linker (molecule II, light blue) shown in Fig. 1e. (**h**) The linker region of molecule I (N125, R126) contacts the catalytic domain surface (G188, T298) of molecule II via side chain specific interactions.

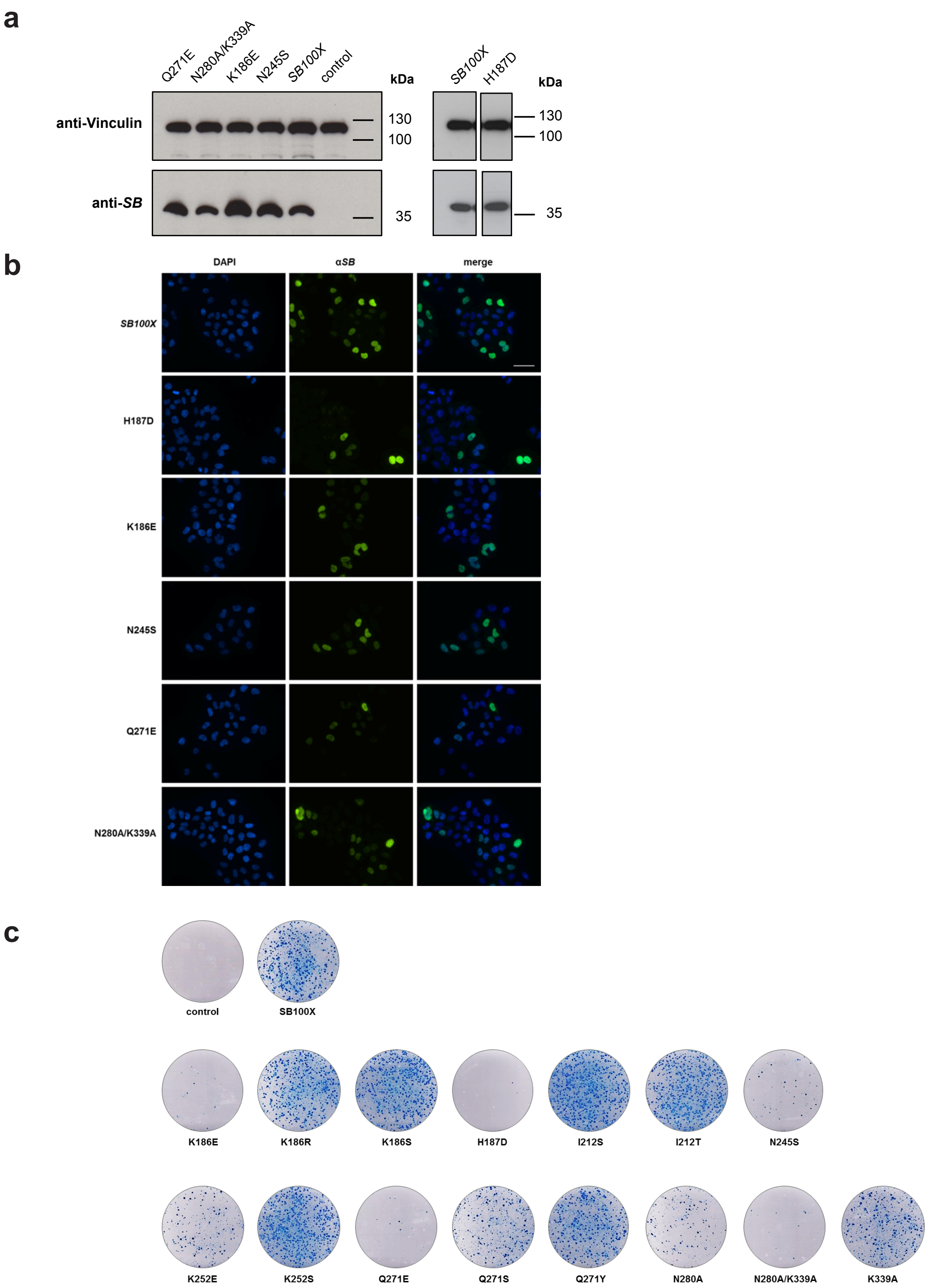

Supplementary Figure 4. *In vivo* transposition assays with mutated SB100X transposases. (**a**) Western blot analysis to confirm expression of the low activity mutants. Vinculin was used as a loading control. The positions of molecular size markers are indicated on the right. The boxes marked 'SB100X' and 'H187D' show two separated lanes from the same blot. (**b**) Immunostaining of HeLa cells to confirm nuclear localization of low activity mutants. Transposase was detected with anti-SB primary antibodies and stained with Alexa 488 secondary antibodies (green). The cell's nuclei were DAPI stained (blue). The scale bar indicates 50 µm. (**c**) Transposition activity assays[4] with all tested SB mutants. HeLa cells were co-transfected with a transposon donor plasmid and the indicated transposase mutant. Cells were selected for puromycin resistance and stained with methylene blue before quantification of viable cell colonies. Viable colonies indicate integration of a resistance carrying transposase into the HeLa cells' nuclei.

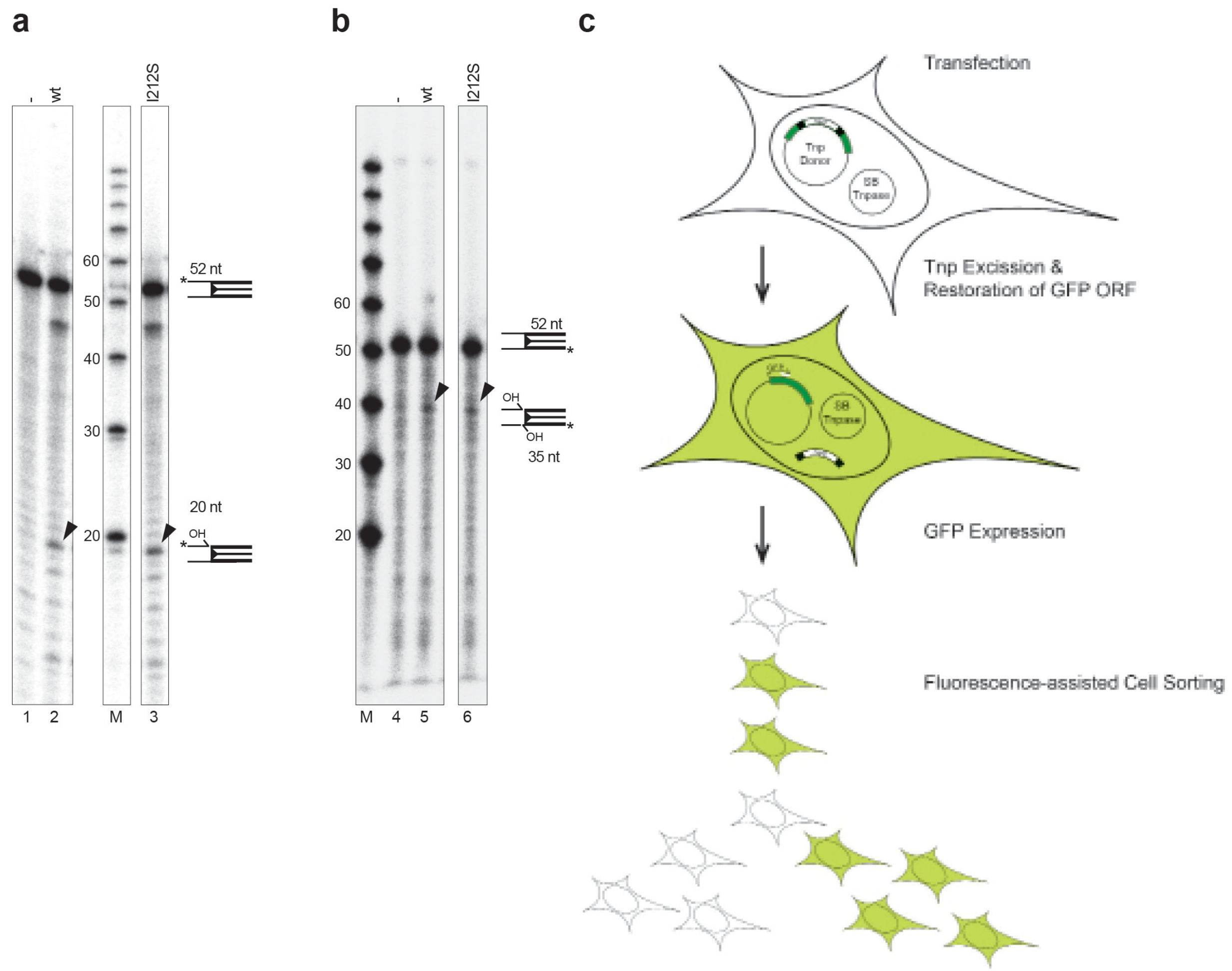

Supplementary Figure 5. Excision assays with SB100X and I212S transposase mutants. (**a**) *In vitro* excision assay monitoring non-transferred strand (NTS) cleavage. The 52 nt tnp end substrate (^{32}P-labeled on NTS, illustrated by cartoons next to gel image) is separated from reaction products on 12% Urea PAGE gel. Black arrowheads indicate specific cleavage products (20 nt). Markers (M) were run next to the protein containing lanes and bands are labeled next to the gel images. Controls (-) did not contain SB100X transposase. (**b**) *In vitro* excision assay monitoring transferred strand (TS) cleavage. For identification of the TS cleavage products (35 nt), the tnp end substrate was ^{32}P-labeled on the TS. Figure labels as in (**a**). (**c**) Schematic representation of the *in vivo* excision assay. HeLa cells are co-transfected with tnp donor- and transposase expression plasmids (SB Tnpase). Precise excision of the *SB* tnp restores the GFP ORF, resulting in green fluorescent cells that can be detected and quantified by FACS analysis.

REFERENCES

1. Richardson JM, Colloms SD, Finnegan DJ, Walkinshaw MD. Molecular Architecture of the Mos1 Paired-End Complex: The Structural Basis of DNA Transposition in a Eukaryote. *Cell* **138**, 1096-1108 (2009).

2. Krissinel E, Henrick K. Inference of macromolecular assemblies from crystalline state. *Journal of molecular biology* **372**, 774-797 (2007).

3. Goodwin KD, He H, Imasaki T, Lee S-H, Georgiadis MM. Crystal Structure of the Human Hsmar1-Derived Transposase Domain in the DNA Repair Enzyme Metnase. *Biochemistry* **49**, 5705-5713 (2010).

4. Ivics Z, Hackett PB, Plasterk RH, Izsvak Z. Molecular reconstruction of Sleeping Beauty, a Tc1-like transposon from fish, and its transposition in human cells. *Cell* **91**, 501-510 (1997).
